# Evidence of validity and reliability of the Compressed Speech Test with Figures

**DOI:** 10.1590/2317-1782/20232023065en

**Published:** 2024-03-25

**Authors:** Taissane Rodrigues Sanguebuche, Karina Carlesso Pagliarin, Bruna Pias Peixe, Denis Altieri de Oliveira Moraes, Michele Vargas Garcia

**Affiliations:** 1 Programa de Pós-graduação em Distúrbios da Comunicação Humana, Universidade Federal de Santa Maria - UFSM - Santa Maria (RS), Brasil.; 2 Departamento de Fonoaudiologia, Universidade Federal de Santa Maria - UFSM - Santa Maria (RS), Brasil.

**Keywords:** Test Validity, Child, Speech, Auditory Perception, Auditory Perception Disorders

## Abstract

**Purpose:**

To seek evidence of validity and reliability for the Compressed Speech Test with Figures.

**Methods:**

The study was subdivided into three stages: construct validation, criteria and reliability. All participants were aged between 6:00 and 8:11. For the construct, Compressed Speech with Figures and the gold standard Adapted Compressed Speech test were applied to children with typical phonological development. For criterion analysis, Compressed Speech with Figures was applied in two groups, with typical (G1) and atypical (G2) phonological development. Finally, the application protocols underwent analysis by two Speech Therapists, with experience in the area of Central Auditory Processing, seeking to obtain an inter-evaluator reliability analysis.

**Results:**

The correlation test indicated an almost perfect construct (correlation 0.843 for the right ear and 0.823 for the left ear). In the criterion analysis, it was noticed that both groups presented satisfactory results (G1 = 99.6 to 100%; G2 = 96 to 96.5%). The reliability analysis demonstrated that the protocol is easy to analyze, as both professionals presented unanimous responses.

**Conclusion:**

It was possible to obtain evidence of validity and reliability for the Compressed Speech with Figures instrument. The construct analysis showed that the instrument measures the same variable as the gold standard test, with an almost perfect correlation. In the criterion analysis, both groups presented similar performance, demonstrating that the instrument does not seem to differentiate populations with and without mild phonological disorder. The inter-evaluator reliability analysis demonstrated that the protocol is easy to analyze and score.

## INTRODUCTION

Central Auditory Processing (CAP) refers to a set of specific skills on which the subject depends to understand what they hear^(1)^. More specifically, CAP is the construction made based on the auditory signal, aiming to make the information useful, and cannot be summarized only in the perception of sound, but also in the identification, location, attention, analysis, memorization and retrieval of information^(2)^.

To assess CAP skills, different behavioral tests are needed, which simulate challenging everyday situations. The complete battery must consist of low-redundancy monaural tests, dichotic tests, temporal processing tests and binaural interaction tests^(3)^.

In low-redundancy monaural tests, the target category of this study, there is an extrinsic reduction of the speech signal, through modification of frequency, time and intensity. However, until then, all standardized tests in this category required verbal responses^(4)^, which became an obstacle in the assessment of this auditory ability in children with speech sound disorders, for example, since it was not possible to decipher whether the error came from a lack of sound discrimination or unintelligible speech production. Thus, the need to construct a new test, based on figures, was understood^(5)^.

The development of an instrument in any area of health requires immersion in Psychometrics, to analyze whether it really measures what it proposes and whether its answers are reliable^(6)^. The international literature still emphasizes that only after validity and reliability studies should the materials be made available for use, whether in clinical or scientific practice^(7)^.

Therefore, after the test development stage^(5)^, as psychometric studies suggest, the instrument was applied to a pilot sample, demonstrating its easy and quick applicability. Thus, the objective of this study was to continue the validation process of the Compressed Speech with Figures instrument^(5)^, seeking evidence of validity criterion, construct and reliability in children with typical phonological development and phonological disorders.

## METHODS

### Ethical aspects

This study is quantitative and cross-sectional in nature. It followed all ethical precepts in accordance with resolution 510/16 of the National Health Council. Collection began after approval by the Research Ethics Committee, under number 5,197,934, which required all participants to consent to voluntary participation and sign the Free and Informed Consent Form (FICF) or Term of Assent, in which, contained information about the procedures performed, risks, benefits and confidentiality of research data. All assessments were performed at the Audiology Outpatient Clinic of a higher education institution.

### Participants and procedures

The total sample consisted of 30 subjects, of both sexes, aged between 6:00 and 8:11, who were recruited by convenience. The age group was selected based on studies that consider phonological disorder up to nine years of age, being called residual speech error after this period^(8)^, as well as studies that suggest early investigation of central auditory abilities such as predictive factor for speech development^(1,9)^. The subjects underwent the following procedures:

1. Visual inspection of the external acoustic meatus, using a Mikatos otoscope;
2. Pure Tone Threshold Audiometry and Logoaudiometry, using the Interacoustics brand audiometer, model AD229e;
3. Acoustic Immittance Measurements, using the Interacoustics brand Immittance Meter, model AT235;
4. Orofacial Myofunctional Assessment with Score (OMES)^(10)^, seeking to identify alterations in the phonoarticulatory organs and the functions of the stomatognathic system that could interfere with the correct production of sounds;
5. Phonological Assessment Instrument (INFONO)^(11)^, seeking to analyze the presence of phonological disorders, through the application of the picture naming stage.

All children should have hearing thresholds within normal limits bilaterally, that is, 15 dB up to seven years of age^(12)^ and up to 20 dB for older ages^(13)^; Logoaudiometry compatible with audibility thresholds; type A tympanometric curves, that is, the maximum point of compliance occurs between +100 and -100 daPa and the amplitude between 0.3 and 1.6 ml^(14)^; contralateral acoustic reflexes present at frequencies of 0.5, 1, 2 and 4 kHz bilaterally; normality in the Orofacial Myofunctional Examination^(10)^. Subjects who presented evident neurological and/or psychological alterations, phonetic disorders, malformations and congenital orofacial syndromes were excluded from the sample. It is worth noting that all assessments were performed in a single day, taking approximately 1 hour and 30 minutes each.

The INFONO result was decisive for the distribution of the sample. Group 1 (G1) was composed of 22 children with typical phonological development, who responded to the Adapted Compressed Speech (ACS)^(15)^, gold standard, and Compressed Speech with Figures (CSF)^(5)^ tests. Group 2 (G2) was made up of eight children with atypical phonological development, who responded only to the CSF test, due to their speech disorder.

Chart 1 presents the description of the participants regarding gender and age.

**Chart 1:** Description of the sample regarding gender and age

	Typical group (G1)	Atypical group (G2)
**N**	22	8
**Gender M/F**	11/11	4/4
**Age (average)**	7.26	7

**Caption:** G1 = Group 1; G2 = Group 2; N = number of subjects; M = male; F = female

The study was performed in three stages: 1. Construct validation; 2. Criterion validation; 3. Reliability. Chart 2 presents a description of the participants and selection criteria for each stage of the research.

**Chart 2:** Description of participants and selection criteria

Stages	Subjects	Selection criteria
Stage 1. Construct validation	22 typical children	Children aged between 6:00 and 8:11 with typical phonological development.
Stage 2. Criterion validation	22 typical children and 8 atypical children	Children aged between 6:00 and 8:11, with typical and atypical phonological development.
Stage 3. Reliability analysis (inter-evaluator)	02 speech therapists	Speech therapists, with clinical experience in the area of CAP.

The ACS test^(15)^ consists of the presentation of two lists with 25 two-syllable words, each, monaurally, compressed 60% of the time. The child was instructed to repeat the word heard in an accelerated manner, in the way he/she understood. The CSF^(5)^ test, despite having the same structure, that is, two lists with 25 two-syllable words each, with 60% time compression, is supported by visual material so that the child, instead of repeating the words heard, can point to the image that represents it. Therefore, during the application, the child remained with the image booklet in hand so that he could answer the test. Both tests were applied with an intensity of 40 dBSL, monaural presentation, after calibration of the AD229e audiometer, using supra-aural headphones.

As mentioned above, initially, construct analysis was performed based on the responses from G1, through a comparison between ACS and CSF. For this purpose, a Spearman correlation analysis was performed.

Subsequently, criterion analysis was performed by comparing G1 and G2 in the CSF test. A normality analysis was performed for the variables CSF RE (%) and CSF LE (%) using the Kolmogorv-Smirnov test, which rejected the hypothesis (p = 0.001). Therefore, the variables were compared using the non-parametric Wilcoxon test.

Furthermore, after performing the tests, the application protocols were analyzed by two speech therapists, with experience in the CAP area, seeking to obtain an inter-rater reliability analysis. All data were stored and analyzed using SPSS v.22 statistical software, with p ≤ 0.05 being considered significant results.

## RESULTS

Table 1 presents a summary of the variables analyzed for construct validity. The variables ACS right ear (RE) and CSF RE were positively and strongly correlated (Rho=0.843; p ≤0.001), as well as ACS left ear (LE) and CSF LE (Rho=0.823; p ≤0.001). Therefore, it is possible to infer that the instruments evaluate the same variable, carrying an almost perfect correlation and excellent construct validity.

**Table 1:** Correlation between the Adapted Compressed Speech Tests and Compressed Speech with Figures per ear

	N	P-value	Rho
ACS RE	22	≤0.001*	0.843
CSF RE			
ACS LE	22	≤0.001*	0.823
CSF LE			

Statistical test: Spearman

* = statistical significance; Rho = correlation coefficient

**Caption:** ACS = Adapted Compressed Speech; CSF = Compressed Speech with Figures; RE = right ear; LE = left ear; N = number of subjects

For criterion analysis, it is important to highlight that there was no association between gender and groups with typical and atypical phonological development, G1 and G2, respectively (p=0.341). It is also worth noting that all children presented mild phonological disorders.

Table 2 presents the comparison of performance between groups in the CSF test. It is possible to observe that in both groups, the test did not show a significant difference between the percentages of RE and LE. This demonstrates that the instrument does not seem to differentiate populations with and without mild phonological disorders, that is, both groups have similar performance. However, it can be seen that G2 has a slightly lower average number of correct answers than G1.

**Table 2:** Description and comparison of performance between groups with typical (G1) and atypical (G2) phonological development

Group	N	Variable	Mean	SD	P-value
G1	22	CSF RE (%)	100.0	-	0.157
		CSF LE (%)	99.6	1.2	
G2	8	CSF RE (%)	96.5	2.6	0.564
		CSF LE (%)	96.0	2.1	

Statistical test: Wilcoxon

**Caption:** G1 = Group 1; G2 = Group 2; N = number of subjects; CSF = Compressed Speech with Figures; RE = right ear; LE = left ear; SD = standard deviation

In the inter-evaluator reliability analysis, it was not possible to apply statistical comparison tests, since the scoring of the protocols performed by the evaluators were 100% compatible. This demonstrates that the protocol is easy to analyze and score.

## DISCUSSION

The union of language studies and CAP brought great gains for professionals and patients in these areas^(1,9,16)^. It is possible to see an increase in the number of studies that prove that the production of intelligible speech depends both on programming and motor execution capabilities, as well as on the ability to process paradigms of the acoustic process. Therefore, it is clear that there is an intimate relationship between acoustic perception and the production of speech sounds. Today it is clear that delays in the maturation stages of auditory skills can be a predictive factor for disorders in the development of speech and oral language, hence the importance of assessing them early^(1,9,16)^.

A recent study^(17)^ serves as a basis for understanding that nowadays, the procedures adopted in a construction and validation process must be rigorous and based on scientific evidence. International literature^(7,18)^ points out that it is essential that instruments used for diagnostic purposes undergo psychometric validity and reliability studies and suggest that only after these studies should they be made available for use.

In 2004, Rabelo developed the Compressed Speech Test for Brazilian Portuguese, due to the impossibility of evaluating auditory closure ability with time compression in Brazil^(4)^. As mentioned above, the test was widely used in clinical practice and scientific research, however, its application to subjects with speech disorders became a major dilemma, due to the requirement for verbal responses. Therefore, the present study sought, after the content validation stage, to perform construct and criterion validation, as well as reliability analysis to release the instrument to the scientific community and enable new studies on the topic.

Construct validity is related to the degree to which an instrument is measuring the construct of interest. This validity is the most complex and difficult to determine, since it studies the degree to which the measurement scores relate to other scores of conceptually related constructs^(19)^. In the present study, convergent validity was used, that is, the application of a correlation test between the measurements of the proposed instrument and a gold standard test (Table 1). Thus, it can be inferred that the instrument really evaluates what it proposes, due to the strong correlation between the new instrument and the existing test.

Criterion validity seeks to verify whether the instrument is truly capable of detecting alterations^(19)^. The comparison between the results of Compressed Speech with Figures in groups with typical and atypical phonological development demonstrated that even children with mild phonological disorders obtained satisfactory results (Table 2). It is believed that the lack of a statistically significant relationship can be explained, in part, by the constitution of the group of subjects evaluated. In the sample there was no variation in the severity of the phonological disorder, with all children presenting a little altered system.

Furthermore, the test under analysis presents a great difference in relation to existing ones, the visual support material, normally understood as an easier instrument (closed set). An adaptation study that carried out all the validity and reliability stages suggested that its closed set protocol be applied when individuals had low performance in the open set application, also emphasizing this difference between the materials^(20)^. Therefore, it is understood as a limitation of the present study that it was applied to only a sample of children with low difficulty, making it necessary to use the instrument in other populations, with different degrees of phonological disorder and even other associated pathologies.

Furthermore, an instrument is considered reliable when it consistently reproduces the results applied on different occasions or by different evaluators, representing one of the main measurement properties. Therefore, the present study used inter-evaluator analysis as a basis. Some studies^(20,21)^ also considered it essential to analyze the vulnerability of the instrument to sources of error, which constitute threats to the validity of the test. The present study, as well as the studies mentioned above, presented unanimous results among the evaluators and, therefore, reliable.

Thus, following psychometric precepts, it was possible to complete the stages of construct validation, criterion validation and reliability analysis. The authors suggest that, in future studies, the instrument be applied to different samples with different degrees of phonological disorder, pathologies and paired groups, in order to qualify and analyze their responses.

## CONCLUSION

It was possible to obtain evidence of construct, criteria and reliability for the Compressed Speech with Figures instrument. The construct analysis showed that the instrument measures the same variable as the other standard test, with an almost perfect correlation. In the criterion analysis, both groups presented similar performance, demonstrating that the instrument does not seem to differentiate populations with and without mild phonological disorders. The reliability analysis demonstrated that the protocol is easy to analyze and score.

## References

[1] Dillon H, Cameron S (2021). Separating the causes of listening difficulties in children. Ear Hear.

[2] Katz J, Wilde L, Katz J. (1999). Tratado de audiologia clínica..

[3] Martins JH, Alves M, Andrade S, Falé I, Teixeira A (2021). Auditory processing disorder test battery in European Portuguese: development and Normative Data for Pediatric Population. Audiology Res.

[4] Rabelo CM (2004). Processamento Auditivo: Teste de fala comprimida em português em adultos normo-ouvintes..

[5] Sanguebuche TR (2023). Teste de Fala Comprimida com Figuras: construção e validação do instrumento.

[6] Echevarria-Guanilo ME, Gonçalves N, Romaniski PJ (2017). Propriedades psicométricas de instrumentos de medidas: bases conceituais e métodos de avaliação - parte I. Texto Contexto Enferm.

[7] Kirk C, Vigeland L (2014). A psychometric review of norm-referenced tests used to assess phonological error patterns. Lang Speech Hear Serv Sch.

[8] Alexandre PD, Beber BC, Dias RF (2020). Erros Residuais de Fala - estudo preliminar sobre características dos sistemas fonético/fonológico em falantes do Português Brasileiro. Distúrb Comun.

[9] Sahlén B, Brännström KJ, Lyberg Åhlander V, Rudner M (2020). Children listen: psychological and linguistic aspects of listening difficulties during development. Front Psychol.

[10] Felício CM, Folha GA, Gaido AS, Dantas MMM, Azevedo-Marques PM (2014). Protocolo de avaliação miofuncional orofacial com escores informatizado: usabilidade e validade. CoDAS.

[11] Ceron MI, Gubiani MB, Oliveria CR, Keske-Soares M (2020). Instrumento de Avaliação Fonológica (INFONO): estudo piloto. CoDAS.

[12] Northern JL, Downs MP (2002). Hearing in children..

[13] WHO: World Health Organization (2020). Prevention of blindness and deafness.

[14] Jerger J (1970). Clinical experience with impedance audiometry. Arch Otolaryngol.

[15] Folgearini JS, Goulart LLA, Silva DD, Vellozo FF, Mezzomo CL, Garcia MV (2016). Teste de fala comprimida: adaptação e validação. Rev CEFAC.

[16] Marchetti PT, Dalcin LM, Balen AS, Mezzomo CL (2022). Processamento auditivo temporal e os traços distintivos de crianças com transtorno fonológico. Rev CEFAC.

[17] Luís C, Abrantes A, Oliveira C, Alves M, Martins JH (2023). Desenvolvimento e validação de conteúdo de um Programa de Intervenção em Processamento Auditivo para crianças em idade escolar. CoDAS.

[18] Mcleod S, Verdon S. (2014). A review of 30 speech assessments in 19 languages other than English. Am J Speech Lang Pathol.

[19] Polit DF, Yang FM (2016). Measurement and the measurement of change..

[20] Araújo MEB, Lima MCO, Carvalho WLO, Brazorotto JS (2021). Adaptation of the Brazilian Functional Auditory Performance Indicators - Short Version. CoDAS.

[21] Ceron MI, Gubiani MB, Oliveira CR, Keske-Soares M (2018). Evidence of validity and reliability of a phonological assessment tool. CoDAS.

