# Supplementary material for: Evidence of validity and reliability of the Compressed Speech Test with Figures
Source: Codas. 2024 Mar 25;36(2):e20230065. doi: 10.1590/2317-1782/20232023065en (PMC10984583; doi:10.1590/2317-1782/20232023065en)
Supplement: Translation

# Translation: Evidências de validade e fidedignidade do Teste de Fala Comprimida com Figuras

**DOI:** 10.1590/2317-1782/20232023065pt

**Authors:** Taissane Rodrigues Sanguebuche, Karina Carlesso Pagliarin, Bruna Pias Peixe, Denis Altieri de Oliveira Moraes, Michele Vargas Garcia

**Affiliations:** 1 Programa de Pós-graduação em Distúrbios da Comunicação Humana, Universidade Federal de Santa Maria - UFSM - Santa Maria (RS), Brasil.; 2 Departamento de Fonoaudiologia, Universidade Federal de Santa Maria - UFSM - Santa Maria (RS), Brasil.

**Keywords:** Validade dos Testes, Criança, Fala, Percepção Auditiva, Transtornos da Percepção Auditiva

## Abstract

**Objetivo:**

Buscar evidências de validade e fidedignidade para o Teste de Fala Comprimida com Figuras.

**Método:**

O estudo foi subdividido em três etapas: validação de construto, critério e fidedignidade. Todos os participantes tinham idade entre 6:00 e 8:11. Para o construto, aplicou-se o Fala Comprimida com Figuras e o teste padrão ouro Fala Comprimida Adaptado em crianças com desenvolvimento fonológico típico. Para análise de critério, aplicou-se o Fala Comprimida com Figuras em dois grupos, com desenvolvimento fonológico típico (G1) e atípico (G2). Por fim, os protocolos de aplicação passaram pela análise de duas Fonoaudiólogas, com experiência na área do Processamento Auditivo Central, buscando obter uma análise de fidedignidade interavaliadores.

**Resultados:**

O teste de correlação indicou um construto quase perfeito (Rho=0,843 para orelha direita e Rho=0,823 para orelha esquerda). Na análise de critério, percebeu-se que ambos os grupos apresentaram resultados satisfatórios (G1 = 99,6 a 100%; G2 = 96 a 96,5%). Já a análise de fidedignidade demonstrou que o protocolo é de fácil análise, pois ambos os profissionais apresentaram respostas unânimes.

**Conclusão:**

Foi possível obter evidências de validade e fidedignidade para o instrumento de Fala Comprimida com Figuras. A análise de construto evidenciou que o instrumento mede a mesma variável que o teste padrão outro, com correlação quase perfeita. Na análise de critério, ambos os grupos apresentaram desempenho semelhante, demonstrando que o instrumento não parece diferenciar populações com e sem transtorno fonológico leve. A análise de fidedignidade interavaliador demonstrou que o protocolo é de fácil análise e pontuação.

## INTRODUÇÃO

O Processamento Auditivo Central (PAC) refere-se a um conjunto de habilidades específicas das quais o sujeito depende para compreender o que ouve^(1)^. Mais especificamente, o PAC é a construção feita com base no sinal auditivo, visando tornar a informação útil, não podendo ser resumido apenas na percepção do som, mas também na identificação, localização, atenção, análise, memorização e recuperação da informação^(2)^.

Para avaliar as habilidades do PAC são necessários diferentes testes comportamentais, que simulam situações desafiadoras do cotidiano. A bateria completa deve ser composta por testes monoaurais de baixa redundância, dicóticos, processamento temporal e testes de interação binaural^(3)^.

Nos testes monoaurais de baixa redundância, categoria alvo deste estudo, ocorre uma redução extrínseca do sinal de fala, por meio da modificação de frequência, tempo e intensidade. Entretanto, até então, todos os testes padronizados dessa categoria exigiam respostas verbais^(4)^, o que se tornou um obstáculo na avaliação dessa habilidade auditiva em crianças com transtorno dos sons da fala, por exemplo, visto que não era possível decifrar se o erro advinha da falta de discriminação do som ou por uma produção de fala ininteligível. Assim, entendeu-se a necessidade da construção de um novo teste, com base em figuras^(5)^.

A elaboração de um instrumento em qualquer área da saúde exige uma imersão na Psicometria, para analisar se o mesmo realmente mede o que se propõe e se suas respostas são confiáveis^(6)^. A literatura internacional ainda enfatiza que somente após estudos de validade e de fidedignidade os materiais devem ser disponibilizados para uso, seja na prática clínica ou científica^(7)^.

Sendo assim, após a etapa de elaboração do teste^(5)^, como sugerem os estudos psicométricos, o instrumento foi aplicado em uma amostra piloto, demonstrando-se de fácil e rápida aplicabilidade. Assim, o objetivo desse estudo foi dar continuidade ao processo de validação do instrumento de Fala Comprimida com Figuras^(5)^, buscando evidências de validade de critério, construto e fidedignidade em crianças com desenvolvimento fonológico típico e transtorno fonológico.

## MÉTODO

### Aspectos éticos

Este estudo possui caráter quantitativo e transversal. O mesmo seguiu todos os preceitos éticos conforme a resolução 510/16 do Conselho Nacional de Saúde. As coletas iniciaram após a aprovação pelo Comitê de Ética em Pesquisa, sob o número 5.197.934, o qual exigia que todos os participantes assentissem a participação voluntária e assinassem o Termo de Consentimento Livre e Esclarecido (TCLE) ou Termo de Assentimento, no qual, continham informações sobre os procedimentos realizados, riscos, benefícios e confidencialidade dos dados da pesquisa. Todas as avaliações foram realizadas no Ambulatório de Audiologia de uma instituição de ensino superior.

### Participantes e procedimentos

A amostra total foi composta por 30 sujeitos, de ambos os sexos, com idade entre 6:00 e 8:11, os quais foram recrutados por conveniência. A faixa-etária foi selecionada com base em estudos que consideram o transtorno fonológico até os nove anos de idade, sendo denominado erro residual de fala após tal período^(8)^, assim como, os estudos que sugerem a investigação precoce das habilidades auditivas centrais como fator preditivo para o desenvolvimento da fala^(1,9)^. Os sujeitos passaram pelos seguintes procedimentos:

1) Inspeção visual do meato acústico externo, por meio do otoscópio da marca *Mikatos*;

2) Audiometria Tonal Liminar e Logoaudiometria, utilizando o Audiômetro da marca *Interacoustics*, modelo AD229e;

3) Medidas de Imitância Acústica, por meio do Imitanciômetro da marca *Interacoustics*, modelo AT235;

4) Avaliação Miofuncional Orofacial com Escore (AMIOFE)^(10)^, buscando identificar alterações dos órgãos fonoarticulatórios e das funções do sistema estomatognático que pudessem interferir na produção correta dos sons;

5) Instrumento de Avaliação Fonológica (INFONO)^(11)^, buscando analisar a presença de transtorno fonológico, por meio da aplicação da etapa de nomeação de figuras.

Todas as crianças deveriam apresentar limiares auditivos dentro dos padrões de normalidade bilateralmente, isto é, 15 dB até os sete anos de idade^(12)^ e até 20 dB para idades superiores^(13)^; Logoaudiometria compatível com os limiares de audibilidade; curvas timpanométricas tipo A, isto é, ponto máximo de compliância ocorre entre +100 e -100 daPa e a amplitude entre 0,3 e 1,6 ml^(14)^; reflexos acústicos contralaterais presentes nas frequências de 0,5, 1, 2 e 4 kHz bilateralmente; normalidade no Exame Miofuncional Orofacial^(10)^. Sujeitos que apresentaram alterações neurológicas e/ou psicológicas evidentes, desvio fonético, malformações e síndromes congênitas orofaciais foram excluídos da amostra. Cabe ressaltar que todas as avaliações foram realizadas em um único dia, levando cerca de 1 hora e 30 minutos, cada.

O resultado do INFONO foi determinante para a distribuição da amostra. O Grupo 1 (G1) foi composto por 22 crianças com desenvolvimento fonológico típico, as quais responderam aos testes de Fala Comprimida Adaptado (FCA)^(15)^, padrão ouro, e Fala Comprimida com Figuras (FCF)^(5)^. Já o Grupo 2 (G2) foi composto por oito crianças com desenvolvimento fonológico atípico, que responderam apenas ao teste FCF, devido ao transtorno de fala.

O Quadro 1 apresenta a descrição dos participantes quanto ao sexo e à idade.

**Quadro 1:** Descrição da amostra quanto a sexo e idade

	Grupo típico (G1)	Grupo atípico (G2)
**N**	22	8
**Sexo M/F**	11/11	4/4
**Idade (média)**	7,26	7

**Legenda:** G1 = Grupo 1; G2 = Grupo 2; N = número de sujeitos; M = masculino; F = feminino

O estudo foi realizado em três etapas: 1. Validação de construto; 2. Validação de critério; 3. Fidedignidade. O Quadro 2 apresenta a descrição dos participantes e critérios de seleção para cada etapa da pesquisa.

**Quadro 2:** Descrição dos participantes e critérios de seleção

Etapas	Sujeitos	Critérios de seleção
Etapa 1. Validação de construto	22 crianças típicas	Crianças com idade entre 6:00 e 8:11 com desenvolvimento fonológico típico.
Etapa 2. Validação de critério	22 crianças típicas e 8 crianças atípicas	Crianças com idade entre 6:00 e 8:11, com desenvolvimento fonológico típico e atípico.
Etapa 3. Análise de fidedignidade (interavaliadores)	02 fonoaudiólogos	Fonoaudiólogos, com experiência clínica na área de PAC.

O teste FCA^(15)^ consiste na apresentação de duas listas com 25 palavras dissílabas, cada, de modo monoaural, comprimidas em 60% do tempo. A criança foi orientada a repetir a palavra ouvida aceleradamente, do modo que entendesse. Já o teste FCF^(5)^, apesar de possuir a mesma estrutura, isto é, duas listas com 25 palavras dissílabas cada, com compressão de tempo em 60%, possui o apoio de material visual para que a criança, ao invés de repetir as palavras ouvidas, possa apontar a imagem que a representa. Portanto, durante a aplicação, a criança permaneceu com o livreto de imagens em mãos para que pudesse responder ao teste. Ambos os testes foram aplicados com intensidade de 40 dB NS, apresentação monoaural, após calibração do audiômetro AD229e, utilizando fones supra-aurais.

Como supracitado, inicialmente, foi realizada a análise de construto com base nas respostas do G1, por meio de uma comparação entre FCA e FCF. Para tanto, foi realizada uma análise de correlação de Spearman.

Posteriormente, realizou-se a análise de critério através da comparação entre G1 e G2 no teste FCF. Foi realizada uma análise de normalidade para as variáveis FCF OD (%) e FCF OE (%) por meio do teste de Kolmogorov-Smirnov, o qual rejeitou a hipótese (p = 0,001). Portanto, as variáveis foram comparadas através do teste não-paramétrico de Wilcoxon.

Ainda, após realização dos testes, os protocolos de aplicação passaram pela análise de duas Fonoaudiólogas, com experiência na área do PAC, buscando obter uma análise de fidedignidade interavaliadores. Todos os dados foram armazenados e analisados através do software estatístico SPSS v.22, sendo considerados resultados significativos p ≤ 0,05.

## RESULTADOS

A Tabela 1 apresenta um resumo das variáveis analisadas quanto à validade de construto. As variáveis FCA orelha direita (OD) e FCF OD apresentaram-se positiva e fortemente correlacionadas (Rho=0,843; p ≤0,001), bem como FCA orelha esquerda (OE) e FCF OE (Rho=0,823; p ≤0,001). Portanto, é possível inferir que os instrumentos avaliam a mesma variável, carregando uma correlação quase perfeita e excelente validade de construto.

**Tabela 1:** Correlação entre os Testes de Fala Comprimida Adaptado e Fala Comprimida com Figuras por orelha

	N	P-valor	Rho
**FCA OD**	22	≤0,001^*^	0,843
**FCF OD**			
**FCA OE**	22	≤0,001^*^	0,823
**FCF OE**			

Teste estatístico: Spearman

* = significância estatística; Rho = coeficiente de correlação

**Legenda:** FCA = Fala Comprimida Adaptado; FCF = Fala Comprimida com Figuras; OD = orelha direita; OE = orelha esquerda; N = número de sujeitos

Para análise de critério, é importante salientar que não houve associação entre o sexo e os grupos com desenvolvimento fonológico típico e atípico, G1 e G2, respectivamente (p=0,341). Cabe ressaltar ainda que todas as crianças apresentaram transtorno fonológico de grau leve.

A Tabela 2 apresenta a comparação do desempenho entre os grupos no teste FCF. É possível observar que em ambos os grupos, o teste não apontou diferença significativa entre os percentuais de OD e OE. Isso demonstra que o instrumento não parece diferenciar populações com e sem transtorno fonológico leve, isto é, ambos os grupos têm desempenho semelhante. No entanto, percebe-se que o G2 apresenta média de acertos um pouco menor que o G1.

**Tabela 2:** Descrição e comparação de desempenho entre os grupos com desenvolvimento fonológico típico (G1) e atípico (G2)

**Grupo**	**N**	**Variável**	**Média**	**DP**	**P-valor**
**G1**	22	FCF OD (%)	100,0	-	0,157
		FCF OE (%)	99,6	1,2	
**G2**	8	FCF OD (%)	96,5	2,6	0,564
		FCF OE (%)	96,0	2,1	

Teste estatístico: Wilcoxon

**Legenda:** G1 = Grupo 1; G2 = Grupo 2; N = número de sujeitos; FCF = Fala Comprimida com Figuras; OD = orelha direita; OE = orelha esquerda; DP = desvio padrão

Na análise de fidedignidade interavaliador não foi possível aplicar testes estatísticos de comparação, visto que a pontuação dos protocolos realizados pelos avaliadores foram 100% compatíveis. Isso demonstra que o protocolo é de fácil análise e pontuação.

## DISCUSSÃO

A união dos estudos de linguagem e PAC trouxe grandes ganhos para os profissionais e pacientes das áreas^(1,9,16)^. É possível visualizar o aumento no número de estudos que comprovam que a produção de uma fala inteligível depende tanto das capacidades de programação e execução motora, como também da habilidade de processar os paradigmas do processo acústico. Sendo assim, percebe-se a existência de uma íntima relação entre a percepção acústica e a produção dos sons da fala. Hoje é claro que o atraso nas etapas de maturação das habilidades auditivas podem ser um fator preditivo dos transtornos no desenvolvimento da fala e da linguagem oral, por isso a importância de avaliá-las precocemente^(1,9,16)^.

Um recente estudo^(17)^ serve como base para compreender que nos dias atuais, obrigatoriamente, os procedimentos adotados em um processo de construção e validação devem ser rigorosos e baseados em evidências científicas. A literatura internacional^(7,18)^ aponta como essencial que instrumentos utilizados para fins de diagnóstico passem por estudos psicométricos de validade e fidedignidade e, sugerem, que somente após esses estudos, devem ser disponibilizados para uso.

Em 2004, Rabelo elaborou o Teste de Fala Comprimida para o português brasileiro, devido a impossibilidade de avaliar a habilidade auditiva de fechamento com compressão de tempo no Brasil^(4)^. Como supracitado, o teste foi amplamente utilizado na prática clínica e em pesquisas científicas, porém, tornou-se um grande dilema a aplicação em sujeitos com transtorno de fala, devido a exigência de respostas verbais. Portanto, o presente estudo buscou, após etapa de validação de conteúdo, realizar validação de construto e critério, assim como, análise fidedignidade para liberar o instrumento ao meio científico e possibilitar novos estudos sobre o tema.

A validade de construto está relacionada ao grau em que um instrumento está medindo o construto de interesse. Essa validade é a mais complexa e difícil de ser determinada, uma vez que estuda o grau em que as pontuações da medida se relacionam com outras pontuações de construtos conceitualmente relacionados^(19)^. No presente estudo, fez-se uso da validade convergente, isto é, aplicação de teste de correlação entre as medidas do instrumento proposto e um teste padrão ouro (Tabela 1). Assim, pode-se inferir que o instrumento realmente avalia o que se propõe, devido à forte correlação entre o novo instrumento e o teste já existente.

Já a validade de critério busca verificar se o instrumento realmente é capaz de detectar alterações^(19)^. A comparação entre os resultados do Fala Comprimida com Figuras nos grupos com desenvolvimento fonológico típico e atípico demonstrou que até mesmo crianças com transtorno fonológico leve obtiveram resultados satisfatórios (Tabela 2). Acredita-se que a falta de relação estatisticamente significante pode ser explicada, em parte, pela constituição do grupo de sujeitos avaliados. Na amostra não houve variação na gravidade do transtorno fonológico, tendo todas as crianças apresentado um sistema pouco alterado.

Além disso, o teste em análise apresenta um grande diferencial em relação aos já existentes, o material visual de apoio, normalmente compreendido como um instrumento mais fácil (conjunto fechado). Um estudo de adaptação que realizou todas as etapas de validade e fidedignidade sugeriu que o seu protocolo em conjunto fechado fosse aplicado quando os indivíduos obtivessem baixo desempenho na aplicação em conjunto aberto, já enfatizando também essa diferença entre os materiais^(20)^. Portanto, entende-se como uma limitação do presente estudo a aplicação em apenas uma amostra de crianças com baixa dificuldade, sendo necessário o uso do instrumento em outras populações, com diferentes graus de transtorno fonológico e até mesmo outras patologias associadas.

Ademais, um instrumento é considerado fidedigno quando reproduz de forma consistente os resultados aplicados em diferentes ocasiões ou por diferentes avaliadores, representando uma das principais propriedades de medida. Sendo assim, o presente estudo utilizou como base a análise interavaliador. Alguns estudos^(20,21)^ também consideraram fundamental analisar a vulnerabilidade do instrumento às fontes de erro, que constituem ameaças à validade do teste. O presente estudo, assim como os estudos supracitados, apresentou resultados unânimes entre os avaliadores e, portanto, confiáveis.

Assim, seguindo os preceitos psicométricos, foi possível finalizar as etapas de validação de construto, validação de critério e análise de fidedignidade. As autoras sugerem que, em futuros estudos, o instrumento seja aplicado em diferentes amostras com diferentes graus de transtorno fonológico, patologias e grupos pareados, a fim de qualificar e analisar suas respostas.

## CONCLUSÃO

Foi possível obter evidências de construto, de critério e de fidedignidade para o instrumento de Fala Comprimida com Figuras. A análise de construto evidenciou que o instrumento mede a mesma variável que o teste padrão ouro, com correlação quase perfeita. Na análise de critério, ambos os grupos apresentaram desempenho semelhante, demonstrando que o instrumento não parece diferenciar populações com e sem transtorno fonológico leve. Já a análise de fidedignidade demonstrou que o protocolo é de fácil análise e pontuação.
